# 1363. Moving from Public Health Outbreak Response to Mitigation for a Regional Outbreak of Highly-Resistant New Delhi Metallo-β-Lactamase-Producing *Acinetobacter baumannii* in California, May 2020 – April 2022

**DOI:** 10.1093/ofid/ofac492.1192

**Published:** 2022-12-15

**Authors:** Diana Holden, Tisha Mitsunaga, Shantala Ahanya, Kristy Trausch, Vikram Haridass, Rachel Levit, Kiara Velasquez, Erin Garcia, Neha Sardana, Ashya Cabral, Elias Geraldo Garcia, Emily C Schneider, Erin Epson

**Affiliations:** California Department of Public Health, Richmond, California; California Department of Public Health, Richmond, California; California Department of Public Health, Richmond, California; California Department of Public Health, Richmond, California; California Department of Public Health, Richmond, California; California Department of Public Health, Richmond, California; California Department of Public Health, Richmond, California; California Department of Public Health, Richmond, California; California Department of Public Health, Richmond, California; California Department of Public Health, Richmond, California; California Department of Public Health, Richmond, California; Washington State Department of Health, Shoreline, Washington; California Department of Public Health, Richmond, California

## Abstract

**Background:**

Since May 2020, the California Department of Public Health and 12 local health jurisdictions (LHJ) have responded to a regional outbreak of highly-resistant New Delhi metallo-β-lactamase-producing *Acinetobacter baumannii* (NDM AB). Starting in October 2021, public health shifted from outbreak response to resource-conscious long-term mitigation in affected healthcare facilities, and prevention of spread to interconnected facilities.

**Methods:**

We defined a case as a patient identified with an NDM AB clinical isolate, or NDM positive colonization screening and epidemiologic linkage. In October 2021, we lengthened the screening interval and changed the screening population in outbreak skilled nursing facilities (SNF) and long-term acute care hospitals to only include high-risk patients (total dependence for activities of daily living, having wounds or indwelling devices, or ventilated); we continued to screen ventilator units in ventilator-equipped SNF, and epidemiologically-linked unit(s) in acute care hospitals. Concurrently, public health initiated a prevention collaborative focused on improving environmental services (EVS) practices in the most affected LHJ, including 3 outbreak and 4 non-outbreak SNF to promote sustainable EVS practice improvement and peer-to-peer learning.

**Figure:**
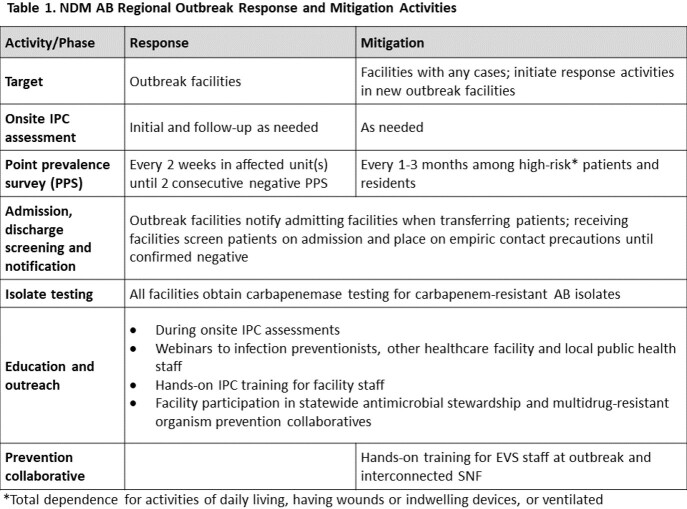

**Results:**

Whereas we identified 170 cases during the outbreak response period May 2020 – September 2021, since October 2021 we identified only 43 cases. Screening test percent positivity decreased from 3.0% (105/3542) through September 2021 to 1.4% (21/1505) since October 2021. During the outbreak response period, we completed 42 infection prevention and control (IPC) onsite assessments that identified poor adherence to core EVS practices; since October 2021, we completed 29 assessments, including 14 for the EVS prevention collaborative.

**Figure:**
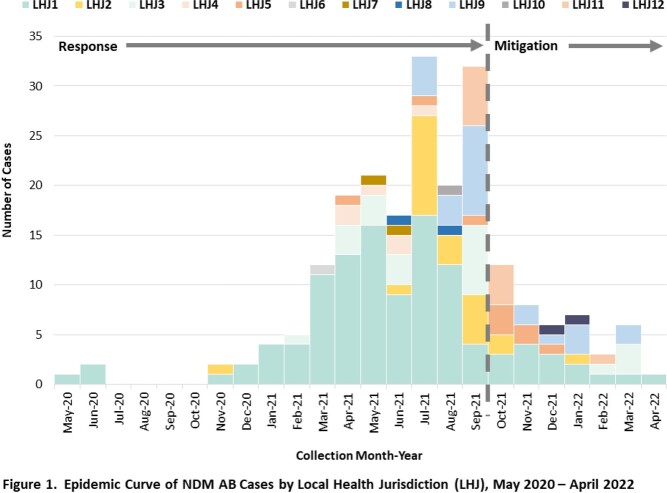

**Figure:**
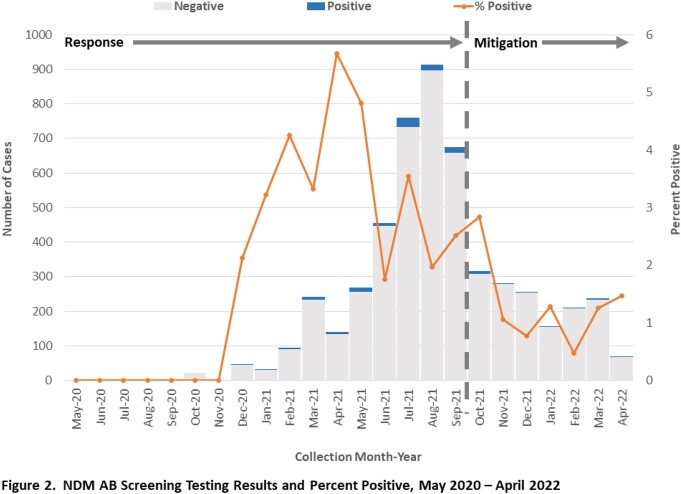

**Conclusion:**

Long-term mitigation of regional, multifacility novel multidrug-resistant organism outbreaks is possible by implementing a coordinated package of interventions including proactive targeted IPC assessment and support at interconnected facilities, and continued routine public health follow-up at outbreak facilities.

**Disclosures:**

**All Authors**: No reported disclosures.

